# *Ligustrum lucidum* W. T. Aiton (broad-leaf privet) demonstrates climatic niche shifts during global-scale invasion

**DOI:** 10.1038/s41598-019-40531-8

**Published:** 2019-03-07

**Authors:** Jaqueline Beatriz Brixner Dreyer, Pedro Higuchi, Ana Carolina Silva

**Affiliations:** Laboratory of Dendrology and Phytosociology, Santa Catarina State University, Agroveterinary Center, Forestry Department, Av Luiz de Camões, 2090, Conta Dinheiro, 88.520-000 Lages, SC Brazil

## Abstract

Biological invasions are a major threat to global biodiversity. *Ligustrum lucidum*, native to temperate Asia, is one of the most invasive plant species in the world. Climate is an important ecological factor influencing species distribution. Therefore, we investigated the climatic niche of *L*. *lucidum* in various regions of the world to determine whether it uses different climatic conditions in its invasive ranges than in its native range. The geographical coordinates of its occurrence were extracted from the Global Biodiversity Information Facility and Southern African Plant Invaders Atlas databases. Climatic variables and altitude data were obtained from WorldClim. We evaluated niche overlap and performed niche similarity tests, and estimated niche shift parameters. *L*. *lucidum* occurs mostly in warm temperate climates. Niche overlap between native and invaded areas was low. Niche similarity tests indicated that the species could expand its occurrence into regions with climates similar to and different from that of its native range. We concluded that *L*. *lucidum* uses different realized climatic niches in its invasive ranges than in its native range. Warmer and wetter climatic conditions may not necessarily constrain this species from establishing populations outside of its native range.

## Introduction

At present, biological invasion is considered a major ecological threat because of its substantial impact on ecosystem function and biodiversity^1^. For this reason, it is useful to elucidate the capacity of a species to shift its climatic niche during invasion. This information may help to predict the ability of an invasive species to expand beyond its geographic range and its responses under different scenarios of global climatic change^2^.

In general, each species has specific environmental requirements to ensure its long-term viability. These conditions are the result of evolutionary history and reflect the fundamental niche in which the species thrives^3^. When many species coexist, the occurrence of each species depends on its realized niche, which is the portion of the fundamental niche in which the species can succeed when biological interactions arise^4^. Climatic factors, such as water availability and temperature, have great impacts on species physiology and survival^5^. They influence the ability of a species to expand to novel areas. Therefore, climatic conditions are considered highly significant ecological determinants of species distribution^6,7^.

During invasion, the shift in realized climatic niche can be evaluated by determining the similarities between the climate of the invaded area and that of the native one^7^. A species may either expand its presence into climatically novel areas or occupy areas that climatically resemble that of its native range^7^. However, the climate type within the native range of the invasive species may be absent or it could remain unoccupied in the invaded area. Some investigators^8–11^ have suggested that exotic species are most likely to invade areas with environmental conditions similar to those found in their native area (i.e., climatic niche stability). Nevertheless, biotic interactions (the release from negative biotic interactions present in the native range and/or positive feedback in the invaded area) and increases in resource availability are important factors favoring biological invasion^12–15^. In fact, investigators have demonstrated that climatic niche shift may also occur^16–19^. These contrasting results reflect the complexity of this research subject and indicate a relative lack of standardized methodologies for data analysis and interpretation^7,20,21^.

At present, *Ligustrum lucidum* W.T. Aiton (broad-leaf privet; Oleaceae) is considered one of the most invasive tree species in the world. It originated in temperate Asia, specifically China^22^, and was introduced into many parts of the world as an urban ornamental^23–31^. Its leaves and fruit may be toxic to humans, and it produces allergenic pollen^32,33^. It is widely distributed globally and has invaded various environments, including degraded sites, conserved areas, wetlands, drier areas, open areas (e.g., fields and plains), and forest understories^24,34^.

Considering the global relevance of *L*. *lucidum* as an invasive species, we aimed to evaluate the climatic niche within its native and invaded ranges. To this end, we determined on a global scale whether *L*. *lucidum* is found in climatic conditions that differ between its invasive ranges and the region of its origin in South Asia. This would demonstrate that its native range only represents a fraction of the elusive fundamental niche of this species.

## Results

**Figure 1:**
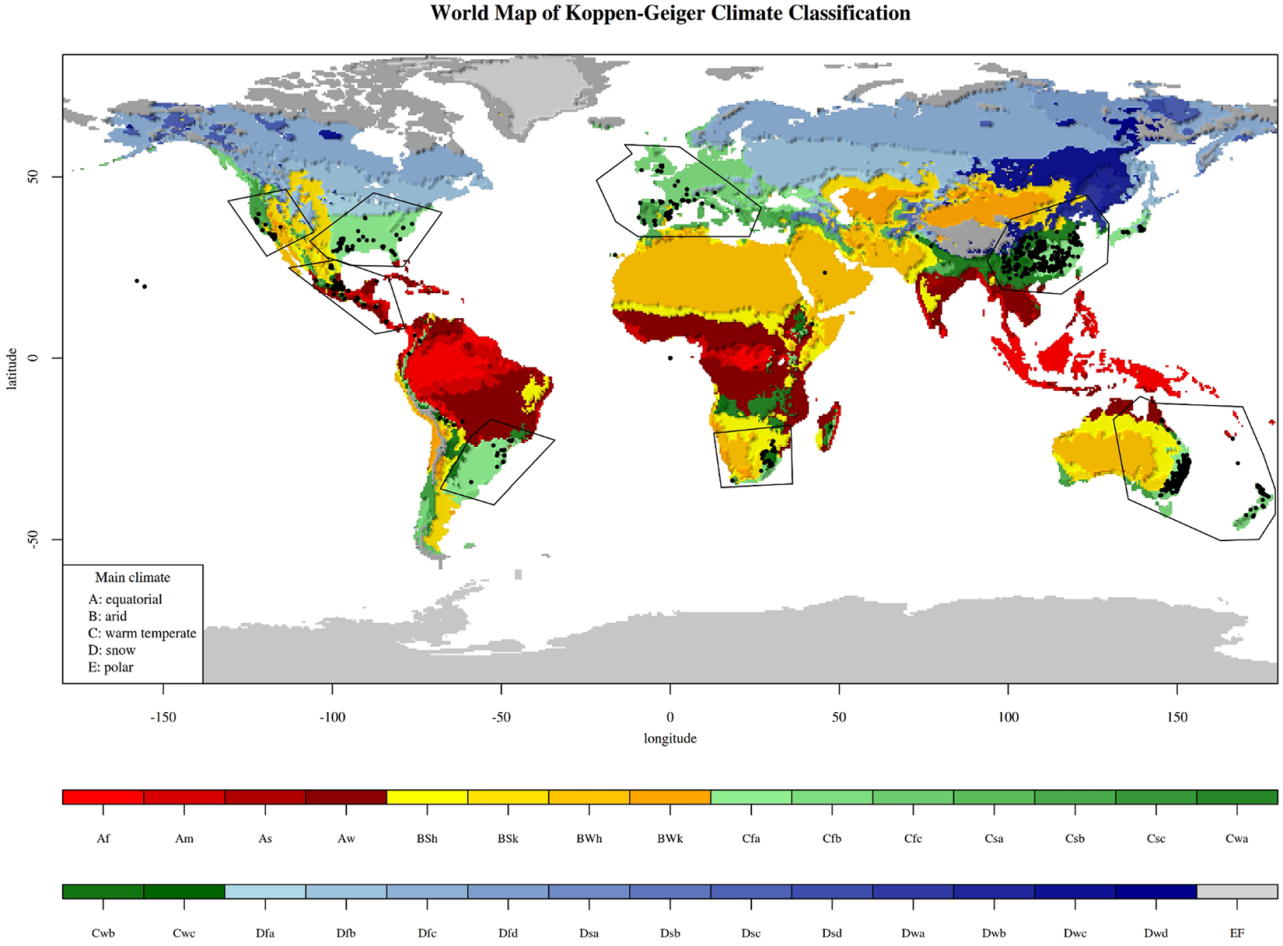
Köppen-Geiger climate classification (1976–2000)^35^ and *Ligustrum lucidum* geographic distribution focusing on the area of its native occurrence in China and other invaded areas for which the climate niche dynamics were evaluated. This figure was generated using R 3.5.1, with code adapted from http://www.rforscience.com/portfolio/koppen-geiger/ ^52^.

*L*. *lucidum* is present on all continents except Antarctica, occurring mostly in warm temperate climates (Köppen-Geiger climate type C) (Fig. 1). Even within the same macroclimate, there was low climatic overlap between the invaded regions of the plant and its native range. The D values varied from 0.00 (Southeastern South America) to 0.12 (Oceania) (Table 1). Except for South Africa and the North American West Coast, the low D values reflected high proportions of expansion (E > 70%) and unfilled (U > 90%) climatic conditions. In South Africa and the West Coast of North America, the expansion values (E %) were relatively low (<35%). Therefore, the climates of these regions were highly similar to those of the native area.

**Table 1:** Climatic niche variation of *Ligustrum lucidum* in relation to its native range (D = niche overlap values; Sim.test = niche similarity test; E = climatic expansion in the invaded area; U = unfilled climatic condition in the invaded area).

Invaded areas	D	Sim.test	E (%)	U (%)	Niche/invasion
Oceania	0.12	0.14	85.17	79.29	D
Europe	0.09	0.06	77.22	96.03	D
Southeastern South America	0.00	1.00	100.00	100.00	D
Central America and Mexico	0.03	0.32	95.87	98.72	D
North American West Coast	0.09	**0**.**02**	31.64	92.66	ND
North American East Coast	0.05	0.08	98.50	99.62	D
South Africa	0.10	0.34	18.82	95.09	D

Numbers in bold have significant *P*-values (≤0.05). D = diverged climatic niche; ND = non-diverged climatic niche.

According to the niche similarity test, however, only the North American West Coast has climatic conditions similar to those in the native range in China (Sim.test: *p* < 0.05). Therefore, the expanded climatic conditions (E %) in this region correspond to the same conditions observed in the native range. However, the climatic conditions of the other invaded areas were dissimilar to those observed in the native range (Sim.test: *p* > 0.05). The climates of the expansion areas (E %) differed significantly from those of the native range. In these cases, *L*. *lucidum* shifted its climatic niche during the invasion process.

**Figure 2:**
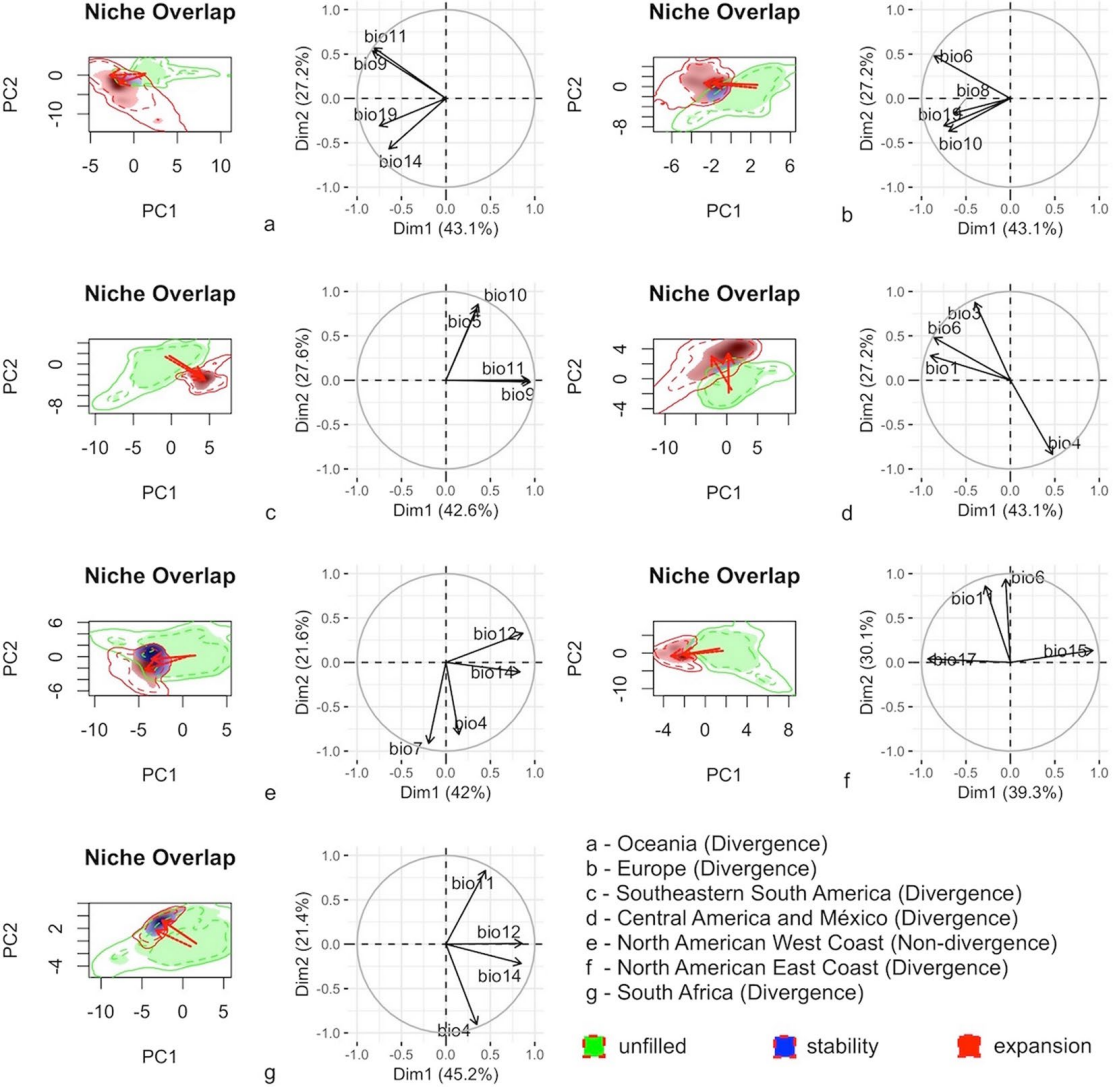
Principal component analysis ordination indicating the climatic variables most relevant to the *Ligustrum lucidum* niche shift during invasion (green = unfilled climatic condition in the invaded area; red = climatic expansion in the invaded area; blue = climatic stability) into different regions (**a** - Oceania; **b** - Europe; **c** - Southeastern South America; **d** - Central America and Mexico; **e** - North American West Coast; **f** - North American East Coast; and **g** - South Africa). The first two axes of each PCA represent the environmental space. The continuous lines represent the available climatic space. The dashed lines represent the species occurrence density. The arrows indicate the change in the centroid from the native range to the invaded area. (bio 1 = Annual Mean Temperature; bio 3: Isothermality; bio 4: Temperature Seasonality (standard deviation ×100); bio 5: Maximum Temperature of the Warmest Month; bio 6: Minimum Temperature of the Coldest Month; bio 7: Annual Temperature Range; bio 8: Mean Temperature of the Wettest Quarter; bio 9: Mean Temperature of the Driest Quarter; bio 10: Mean Temperature of the Warmest Quarter; bio 11: Mean Temperature of the Coldest Quarter; bio 12: Annual Precipitation; bio 14: Precipitation of the Driest Month; bio 15: Precipitation Seasonality; bio 17: Precipitation of Driest Quarter; bio 19: Precipitation of the Coldest Quarter).

For regions in which *L*. *lucidum* shifted its climatic niche (Fig. 2a–g), the first two axes of the multivariate ordinations explained a large proportion of the total inertia (>60%). In general, *L*. *lucidum* expanded its occurrence to warmer and wetter areas (Oceania, Europe, and Southeastern South America) and areas with comparatively lower seasonality of precipitation (North American East Coast) and temperature (Central America and Mexico) than that of the native area.

On the North American West Coast, *L*. *lucidum* occupied the same climatic niche as its native range (Fig. 2e). In this area, expansion occurred under similar climatic conditions. The first two axes of the multivariate ordinations explained a large proportion of the total inertia (66.6%; Axis 1 = 45.2%, and Axis 2 = 21.4%). Figure 2e shows that the climatic conditions of *L*. *lucidum* on the North American West Coast were mainly analogous (blue color) to those in the native distribution area.

## Discussion

*L*. *lucidum* tends to be found within the same type of macroclimate, namely warm temperate^35^. Nevertheless, we showed that it can invade regions with climatic conditions both similar to and different from those found within its native range. Considering that our analysis was conducted using field data that included biotic interactions, this fact suggests that the native range of *L*. *lucidum* only represents a portion of the fundamental niche of the species. In this sense, our study supports the findings of other investigators who demonstrated the potential of species to use different realized climatic niches during invasion^16,36,37^.

A complex interaction among ecological-evolutionary forces such as biological associations, propagule pressure, and adaptive evolution processes^15,38–41^ can explain why an invasive species may not retain the realized niche from its native range. When introduced into a new community, an invasive species may be favored by the lack of negative biological interactions present in its native range and by the potential presence of new mutualists^12–15^. In addition, the repeated introduction of an invasive species may increase propagule pressure^39^ and foster evolutionary processes that lead to invaders with more adaptive potential^40^. Moreover, an invasive species may be preadapted to conditions that are present in the invaded range but are no longer readily available in the native one^4^.

Based on our results and those previously reported, we inferred that the realized niche shifts and worldwide distribution of *L*. *lucidum* are the result of a biological invasion *“perfect storm”* created by intentional human distribution, positive biotic interactions, and strong abiotic plasticity. This species has been widely planted as an ornamental tree by humans^23–26,28–31^. In certain municipalities of southern Brazil, *L*. *lucidum* is the most abundantly planted urban tree^42^. Moreover, the fruit of *L*. *lucidum* may be avidly consumed by local birds^43,44^, which disperse tree propagules in natural areas where they grow rapidly under various ecological conditions^24,34,45^. It is, therefore, evident that *L*. *lucidum* is potentially a serious invasive species.

Overall, our results showed low climatic niche overlap (D) and expansion into areas possessing different climatic conditions (E %). *L*. *lucidum* expanded its presence into more humid and warmer areas with less temperature and precipitation seasonality. Therefore, the species may be tending towards the invasion of wet tropical areas. Tropical ecosystems constitute a vital part of global biodiversity^46^ and the invasion of plant species such as *L*. *lucidum* could place even more pressure on these ecosystems^47^. Examples of these ecosystems can be found in regions in the Brazilian Atlantic Forests, Mesoamerican Forests, East Australian Forests, Madagascar, and the islands of the Indian Ocean. These observations are alarming when one considers biological invasion together with ongoing climate change. An invasive species may be able to thrive under many new environmental conditions in the future^48^.

In conclusion, on a global scale, *L*. *lucidum* has invaded and occupied regions with climates similar to and distinct from that of its native habitat. In Oceania, Europe, Southeastern South America, Central America and Mexico, the North American East Coast, and South Africa, *L*. *lucidum* thrives under various climatic conditions. In these areas, invasion occurred primarily along temperature and precipitation gradients, towards wetter and warmer areas with lower seasonality in temperature and rainfall. This indicates that the native range of *L*. *lucidum* only represents a portion of its fundamental niche. Considering the invasive potential of this species, policies are required to limit or restrict the use, sale, and transport of *L*. *lucidum* outside its native range. In this way, its establishment can be prevented in areas where it has not yet been introduced.

## Methods

### Species geographic occurrence and climatic data

The global occurrence data for *L*. *lucidum* were obtained from georeferenced points in the Global Biodiversity Information Facility^49^ and Southern African Plant Invaders Atlas^50^ databases. Climatic data (1970–2000) were obtained from the WorldClim 2.0 database^51^ at a resolution of 10 min (~20 km). A Köppen-Geiger climate classification map (1976–2000)^35^ was plotted with an R code^52^. Nineteen climatic variables referring to minimum, average, and maximum temperatures and precipitation (bio 1: Annual Mean Temperature; bio 2: Mean Diurnal Range (mean of monthly (max temp − min temp); bio 3: Isothermality (bio 2/bio 7) (×100); bio 4: Temperature Seasonality (standard deviation ×100); bio 5: Maximum Temperature of the Warmest Month; bio 6: Minimum Temperature of the Coldest Month; bio 7: Annual Temperature Range (bio 5–bio 6); bio 8: Mean Temperature of the Wettest Quarter; bio 9: Mean Temperature of the Driest Quarter; bio 10: Mean Temperature of the Warmest Quarter; bio 11: Mean Temperature of the Coldest Quarter; bio 12: Annual Precipitation; bio 13: Precipitation of the Wettest Month; bio 14: Precipitation of the Driest Month; bio 15: Precipitation Seasonality (Coefficient of Variation); bio 16: Precipitation of the Wettest Quarter; bio 17: Precipitation of the Driest Quarter; bio 18: Precipitation of the Warmest Quarter; bio 19: Precipitation of the Coldest Quarter; and alt: Altitude). These variables were used in the data analysis because they are ecologically relevant to species distribution.

### Data analysis

A single occurrence point was filtered for each 10 km × 10 km grid to reduce geographic sampling bias (collections near research centers, universities, herbaria, and others). Polygonal borders were established for regions with significant *L*. *lucidum* occurrence. Eight areas were considered: (1) native tree range in southern China, (2) Oceania, (3) Europe, (4) Southeastern South America, (5) Central America and Mexico, (6) North American West Coast, (7) North American East Coast, and (8) South Africa. Areas without extensive occurrence of this species were excluded to avoid analytical bias.

Considering the native and invaded range climates, principal component analyses (PCAs) were performed pairwise for each study region to investigate variations in realized climatic niche^53,54^. In this way, a two-dimensional climatic species distribution space was created. Ordinations were determined using kernel density estimation for species occurrence^21^. Niche overlap was determined according to Schoener’s D index, which ranges from 0 (no overlap) to 1 (complete overlap).

Niche similarity tests were performed^54^ (α = 0.05) by comparing observed and randomly simulated (n = 100) D values. For this test, an observed D value greater than the randomly simulated D values was considered a null hypothesis, meaning that the invaded niche resembled the native niche; therefore, there was no climate niche shift. We also determined expansion into climatic conditions that are distinct from those in the native range (E) and the unfilled climatic conditions in the native area of the species that are absent in the invaded area (U).

All analyses were carried out using the R statistical programming language v 3.5.1^55^ and the following packages: SDMtools^56^, raster^57^, maptools^58^, ecospat^59^, rgbif^60^, spThin^61^, and splancs^62^.

## Data Availability

The data used in this research are available upon request by contacting the corresponding author.
